# Challenges and Advances in Bioinformatics and Computational Biology

**DOI:** 10.3390/cimb48020185

**Published:** 2026-02-06

**Authors:** Tong Si, Haijun Gong

**Affiliations:** 1 Department of Health and Clinical Outcomes Research, Saint Louis University, St. Louis, MO 63104, USA; 2 Department of Mathematics and Statistics, Saint Louis University, St. Louis, MO 63103, USA

Modern sequencing and high-throughput profiling technologies [1,2] have generated an unprecedented volume of omics data, including single-cell RNA sequencing, genomics, proteomics, metabolomics, and integrated multi-omics measurements. These data enable the systematic investigation of cellular systems at unprecedented resolution, facilitating quantitative characterization of cellular heterogeneity and offering transformative opportunities to deepen our understanding of complex biological processes [3], reconstruct cell type-specific genetic networks [4], elucidate disease mechanisms [5], and accelerate the development of personalized and precision medicine [6].

Despite these opportunities, the scale, complexity, and heterogeneity of modern omics datasets present substantial analytical and computational challenges. Single-cell and multi-omics data are typically high dimensional, sparse, and noisy, with pervasive missing values [7], which limit the effectiveness of traditional statistical and computational approaches. Moreover, the integration of heterogeneous data across multiple modalities and experimental conditions further complicates downstream analysis and biological interpretation. Key open challenges include accurate missing-value imputation [8,9], reliable disease biomarker discovery [10], microbial taxonomy profiling [11], integration of heterogeneous data across multiple omics modalities [12], as well as trajectory inference [13,14] and cell-fate modeling [15], spatial and temporal pattern analysis of brain activity and data [16], time-varying network modeling, and the reconstruction of gene regulatory networks [17] that capture dynamic and context-specific cellular behavior. Addressing these challenges requires the development of robust, scalable, and interpretable computational methods that integrate advanced statistical modeling, machine learning, bioinformatics and computational biology approaches to fully leverage the rich information embedded in modern omics data.

Over the past year, bioinformatics and computational biology have witnessed significant advances, with a wide range of new methods [18,19,20] developed to address some longstanding challenges. These advances have been driven by rapid progress in artificial intelligence, machine learning, deep learning–based generative models [21,22], data science, and emerging quantum computing approaches [23], in combination with classical computational methods, to extract meaningful biological insights from high-dimensional, heterogeneous, and multimodal molecular data. This Special Issue of Current Issues in Molecular Biology, entitled “Challenges and Advances in Bioinformatics and Computational Biology,” presents a diverse collection of studies highlighting recent methodological developments, innovative algorithms, scalable analytical frameworks, and impactful applications at the forefront of bioinformatics and computational biology. Collectively, these contributions tackle critical computational challenges posed by modern omics data and highlight how emerging computational techniques are driving new advances in biological discovery, disease understanding, and the translation of data-driven insights into biomedical applications.

Riera Aroche et al. [24] develops a quantum electrodynamic framework for DNA transcription, modeling DNA as a system of nonlinear quantum circuits within the transcription condensate architecture and characterizing transcription dynamics through Hamiltonian formulations from quantum physics. By representing DNA base pairs as anharmonic qubits analogous to radio-frequency superconducting quantum interference devices (RF-SQUIDs) coupled to cavity modes. This study provides a novel physical–mathematical perspective on quantum information processing in DNA.

Wang et al. [25] introduces f-DyGRN, a novel f-divergence–based framework for dynamic gene regulatory network inference from time-series single-cell RNA-seq data. By integrating first-order Granger causality, regularization techniques, partial correlation analysis, and a moving window strategy, the method captures temporal variations in gene interactions across different developmental stages. Applications to simulated and real THP-1 scRNA-seq datasets demonstrate that f-DyGRN outperforms existing approaches in reconstructing dynamic regulatory networks.

Peng et al. [26] proposes MvAl-MFP, a multi-label active learning framework that leverages multi-view feature representations of peptide sequences to reduce labeling costs while maintaining high predictive performance. By integrating multiple peptide feature views with a query-by-committee strategy based on prediction entropy, the method iteratively selects the most informative unlabeled samples for experimental annotation.

Wu et al. [27] systematically analyzes 34 ICD-related regulatory genes across 33 tumor types, evaluating their expression, genetic and epigenetic alterations, and associations with patient survival. Machine learning–based prognostic models revealed some key biomarkers that mediate tumor–immune interactions. The findings provide a comprehensive view of ICD-related genes and highlight potential targets for predicting immunotherapy response and improving clinical outcomes.

Kim et al. [28] introduces PixelCut, an automated framework for predicting trim positions in 16S rRNA amplicon sequencing reads without requiring hyperparameters or prior biological information. By analyzing per-base quality reports from FastQC and applying computer vision–based character recognition, PixelCut accurately identifies low-quality bases for trimming and is accessible via a user-friendly web application or command-line interface.

Xu et al. [29] identifies potential therapeutic targets of dihydromyricetin (DHM) in hepatocellular carcinoma and developed a three-gene prognostic model (DTYMK, MAPT, UCK2) to stratify patients into high- and low-risk groups. High-risk patients exhibited shorter overall survival, more advanced tumor stages, and an immunosuppressive microenvironment characterized by elevated TIDE scores and increased Treg infiltration. These findings provide a foundation for exploring DHM as a natural adjuvant in individualized cancer immunotherapy.

Hernandez-Montiel et al. [30] systematically analyzes genes associated with economically important traits in domestic pigs by compiling data from 116 studies published between 2000 and 2024 and performing de novo functional bioinformatics analyses. Their studies provide an integrated functional framework to support more effective marker-assisted selection strategies in pig breeding programs.

Together, these studies illustrate how advanced computational and machine learning methods can decode complex biological systems from high-dimensional molecular data. They highlight applications ranging from dynamic gene network inference and peptide function prediction to immunotherapy biomarkers and automated sequencing analysis. Collectively, these works emphasize the power of integrative computational approaches to guide therapeutic strategies and advance personalized medicine.

We sincerely thank the authors for their valuable contributions to this Special Issue, and the reviewers for their thoughtful and constructive evaluations. This Special Issue aims to provide a timely and informative resource for researchers in bioinformatics, computational biology, and related fields. We hope that the collective insights presented here will inspire further innovation and foster continued advances in bioinformatics and computational biology.
